# Altered in Vitro Metabolomic Response of the Human Microbiota to Sweeteners

**DOI:** 10.3390/genes10070535

**Published:** 2019-07-15

**Authors:** Emanuel Vamanu, Diana Pelinescu, Florentina Gatea, Ionela Sârbu

**Affiliations:** 1 Faculty of Biotechnology, University of Agronomic Science and Veterinary Medicine, 59 Marasti blvd, 1 District, 011464 Bucharest, Romania; 2 Department of Genetics, ICUB-Research Institute of the University of Bucharest, 36-46 Bd. M. Kogalniceanu, 5th District, 050107 Bucharest, Romania; 3 Centre of Bioanalysis, National Institute for Biological Sciences, 296 Spl. Independentei, 060031 Bucharest, Romania

**Keywords:** steviol, cyclamate, dysbiosis, short-chain fatty acids, ammonium

## Abstract

Non-nutritive sweeteners represent an ingredient class that directly affects human health, via the development of inflammatory processes that promote chronic diseases related to microbiota dysbiosis. Several in vitro tests were conducted in the static GIS1 simulator. The aim of the study was to highlight the effect of sweeteners on the microbiota pattern of healthy individuals, associated with any alteration in the metabolomic response, through the production of organic acids and ammonium. The immediate effect of the in vitro treatment and the influence of the specific sweetener type on the occurrence of dysbiosis were evaluated by determining the biomarkers of the microbiota response. The presence of the steviol reduced the ammonium level (minimum of 410 mg/L), while the addition of cyclamate and saccharin caused a decrease in the number of microorganisms, in addition to lowering the total quantity of synthesized short-chain fatty acids (SCFAs). The bifidobacteria appeared to decrease below 10^2^ genomes/mL in all the analyzed samples at the end of the in vitro simulation period. Barring the in vitro treatment of steviol, all the sweeteners tested exerted a negative influence on the fermentative profile, resulting in a decline in the fermentative processes, a rise in the colonic pH, and uniformity of the SCFA ratio.

## 1. Introduction

Sweeteners are a versatile food ingredient because of their low caloric content. In recent years, several population groups have begun to use these products, even if they have normal blood sugar levels. Sweetness perception is crucial for an individual’s acceptance of food, and the physiological process depends upon the sweetener (maximum 4 mM concentration) and receptor interactions [1]. Two of the most important steps post intake are represented by the absorption and interaction with the physiological processes in the human body. Sweeteners have even been found in breast milk, and they directly impact the child’s responses to sweet taste during the growth period. Over the long term, this high acceptance of sweet taste determines the incidence of diabetes at very young age [2].

The effect of sweeteners on human health has been extensively explored because of the incidence of obesity and diabetes [3]. The biological effect on the microbiota is significant because the impact of regular consumption helps to explain the progression of degenerative pathologies or cancer [4]. From recent studies, it is evident that a direct relationship exists between sweetener consumption, the establishment of dysbiosis, and the development of neurodegenerative diseases [5]. Setting up pre-diabetes is favored by the interaction of the microbiota with different types of sweeteners, which are increasingly being used in food [6]. Understanding the initiation of dysbiosis and pre-diabetic prognosis necessitates a metabolomic approach, as a modern preclinical study method [7]. The physiological mechanism is a reduction in the time of insulin sensitivity, which once initiated has a linear progression until the pathology is established and manifested simultaneously with an increase in body weight [8].

Colonic pH variation could be associated with a reduction in insulin sensitivity—an important feature in people diagnosed with type 2 diabetes. The regular consumption of sweeteners causes the increased incidence of this pathology even at an early age, with the establishment of dysbiosis. A biomarker was considered to be the *Bacteroides* strain level as an indicator of the ability of the microbiota to control insulin resistance. The level of sweetener consumption is an essential factor that is capable of disrupting the functional plasticity of the microbiota, and reflects a clinical risk factor [9].

In vitro studies in simulation systems are a viable alternative to in vivo assays. Dynamic simulation aims at involving the entire physiological system in the digestion of the samples, as well as the absorption of the essential nutrients. The static GIS1—Phase 2 system was adapted for the in vitro dynamic transit, SHIME (Simulator of the Human Intestinal Microbial Ecosystem) being an accepted example in the scientific community [10]. Previous studies [11] have reported that the modulating response of the microbiota in this case was similar to that in vivo. The results revealed the modulation ability of both the microbial and the metabolomic patterns [11]. This study intended to establish the effect of the sweeteners on the microbiota pattern of healthy individuals, associated with alterations in the metabolomic response, through the production of organic acids and ammonium. Untreated healthy microbiota were used as the control to compare the affected pattern that was altered by the in vitro treatment of different sweeteners.

## 2. Materials and Methods 

### 2.1. Chemicals

The reagents used were all of analytical grade (purity > 98%): DL-lactic acid and butyric acid purchased from Fluka (Buchs, Switzerland), acetic acid from Riedel-de-Haën (Seelze, Germany), L-(+)-tartaric acid, formic, citric acid, benzoic acid, succinic acid, malic acid, propionic acid, DL-*p*-hydroxyphenyllactic acid (HO-PLA), sodium hydroxide, sodium chloride, glycerol, and phenyllactic acid (PLA) bought from Sigma-Aldrich (Germany). Phosphoric acid 85% and oxalic acid were supplied by Merck (Hamburg, Germany), cetyltrimethylammonium bromide (CTAB) from LOBA Chemie (Fischamend, Austria), HPLC-grade water and 0.1 and 1 N sodium hydroxide solutions were purchased from Agilent Technologies (USA). All solvents (Merck, Darmstadt, Germany) and solutions were filtered through 0.2-μm membranes (Millipore, Bedford, MA, USA). Peptone water and MRS broth media were purchased from Oxoid Ltd. (Hampshire, UK).

### 2.2. Obtaining the Sweetener Samples

Samples from eight of the most popular sweeteners marketed by different producers were locally purchased (Table 1) [12]. Sample concentrations were about 40 mg active substance (more than 90% purity). The sweetener doses were calculated to provide the sweetening equivalent of two tablespoons of sugar (≈9 g). All the samples used were in commercial form and added during the in vitro tests in the ascending phase of the human colon simulations.

### 2.3. GIS1 In Vitro Model

GIS1—Phase 2, developed in the Pharmaceutical Biotechnology Lab in The Faculty of Biotechnology, UASVM Bucharest, is an in vitro static system which simulates the transit through the three segments of the human colon (www.gissystems.ro). The system uses a single simulation vessel, a 500 mL Duran borosilicate glass bottle, at a constant operating temperature of 37 °C, with variable pH controlled with 1 M sterile NaOH [10]: ascending colon, pH 6; transverse colon, pH 6.5; descending colon, pH 7.0 [13]. Sterile CO_2_ was introduced via an auxiliary module adapted to the fermentation system, and samples were drawn when the in vitro process culminated from the descending segment. The inoculum was based on stool (feces) samples collected from healthy individuals (both sexes) using peptone water over a 7-day stabilization period. Using a microbial load (fingerprint) the simulated medium was inoculated with the equivalent of two tablespoons of sugar. Inoculation was done concurrently by adding samples (sweeteners) that had been sterilized by filtration and dissolved in 0.9% NaCl.

The microbiome from healthy individuals was reconstituted from the samples (feces), according to the ethical guidelines of UASVM Bucharest (ColHumB Registration number: 1418/23.11.2017; www.colhumb.com). The volunteers (five individuals) were representative of both sexes and had not received treatment with antibiotics or any other interfering drugs over the past 6 months, as these agents could alter the microbiome fingerprint. All the samples were collected in 10% glycerol and stored at −15 °C until use [14]. 

### 2.4. Gut Microbiota Pattern Quantification by qPCR

The total microbial DNA was isolated from 1 mL of the culture using the PureLink Microbiome DNA purification kit (Invitrogen, USA). The concentration and purity of the DNA were measured with NanoDrop 8000 spectrophotometers (ThermoFisher Scientific, USA). Quantification of the number of genome copies of the principally significant microbial groups from among the human gut microbiota (*Enterobacteriaceae* family, *Bacteroides*–*Prevotella*–*Porphyromonas* group, *Lactobacillus*–*Lactococcus*–*Pediococcus* group, Firmicutes phylum, *Bifidobacterium* genus) was done by qPCR using the Applied Biosystem 7900HT Real Time-PCR system. Employing a pair of universal primers for the prokaryotes, the bacterial content in each sample was determined. Bacterial quantification was done by developing standard curves using serial dilutions of a known genomic DNA concentration corresponding to *Escherichia* (*E.*) *coli* ATCC 10536, *Lactobacillus* (*L.*) *plantarum* ATCC 8014, *Bifidobacterium* (*B.*) *breve* ATCC 15700, *Enterococcus* (*E.*) *faecalis* ATCC 29,212, and *B. fragilis* DSM2151. The mass of genomic DNA was converted in copy number of 16S rRNA gene according to the Applied Biosystems guide. The Power SyberGreen PCR Master Mix 2X (Applied Biosystems, USA), and 40 ng total DNA were introduced into a qPCR reaction with a 20 µL volume. The amplification conditions were 95 °C for 10 min followed by 40 cycles with 95 °C for 15 s and 60 °C for 60 s. Table 2 shows the sequence of the primers and prevailing conditions [14]. 

### 2.5. Organic Acids and Ammonia Quantification by Capillary Electrophoresis (CE)

The Ammonium Quanto fix kit (Macherey-Nagel GmbH & Co. KG, Duren, Germany) was used to determine the quantity of ammonia [14].

The samples obtained after the in vitro simulation of the large intestine transit were centrifuged at 5000× *g* at 4 °C for 10 min. The supernatants were then collected and filtered through a 0.22-µm pore size filter (Millipore, Bedford, MA, USA) [14]. 

The organic acids were then separated by the capillary electrophoresis method developed earlier using an Agilent CE instrument with a DAD detector and CE standard bare fused-silica capillary (Agilent Technologies, Santa Clara, CA, USA) of 50 μm internal diameter and 72 cm total length (63 cm effective length) [18]. The CE technique employed here falls under the reversed polarity category, the operating conditions being an applied voltage of −20 kV; UV detection was done at 200 nm (direct detection); sample injection was accomplished in hydrodynamic mode, 35 mbar/12 s, maintaining the capillary at 25 °C constant temperature [19]. The background electrolyte used contained 0.5 M of H_3_PO_4_ and 0.5 mM of CTAB as the cationic surfactant (pH adjusted with NaOH to 6.24) and 15% methanol as the organic modifier. Filtration was performed through 0.2-μm membranes (Millipore, Bedford, MA, USA) and degassing was done before use. The order of elution of the organic acids was as follows: formic, oxalic, succinic, malic, tartaric, acetic, citric, propionic, lactic, butyric, benzoic, PLA, and HO-PLA acids, in 20 min analysis time. The capillary was flushed between runs with 0.1 M NaOH for 2 min, H_2_O for 2 min, and the background electrolyte for 4 min [14].

### 2.6. Statistical Analysis

All the parameters investigated were evaluated in triplicate, and the results were expressed as the mean ± standard deviation (SD) values of three observations. The mean and SD values were calculated using the IBM SPSS Statistics 23 software package (IBM Corporation, Armonk, NY, USA). The significance level for the calculations was set as follows: significant, *p* ≤ 0.05; very significant, *p* ≤ 0.01; and highly significant, *p* ≤ 0.001, using the normal distribution of the variables. The differences were analyzed by ANOVA followed by a Tukey post hoc analysis. Analysis and correlation of the experimental data were done with the IBM SPSS Statistics software package (IBM Corporation, Armonk, NY, USA) [14].

## 3. Results

### 3.1. Alteration in the Metabolomic Pattern Post Sweetener In Vitro Treatment

**Figure 1:**
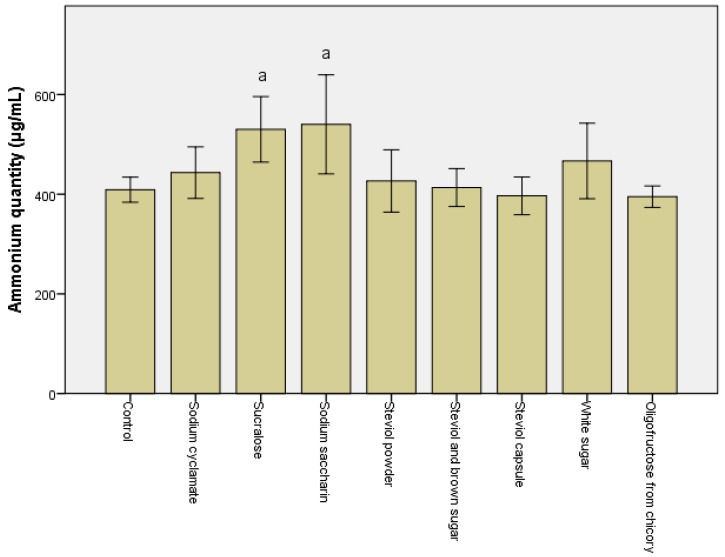
Quantity of ammonium (µg/mL) obtained after the in vitro tests through GIS1 as a measure of the impact of consuming sweeteners on the metabolic activity of the microbiota. Different letters indicate significant statistical differences (a: *p* ≤ 0.05; b: *p* ≤ 0.01; c: *p* ≤ 0.001), *n* = 3.

The quantity of ammonia synthesized is the crucial factor in microbiota modulation. A significant drop (*p* < 0.05) in the ammonia was noted after the in vitro treatment with steviol and oligofructose from chicory-containing sweeteners (Figure 1). In all the other cases, a minimum 10% increase was recorded for all samples, particularly for sucralose and sodium saccharin, with their passage through the descending colon (data not shown).

In vitro sweetener treatment, including those chemically synthesized, exerted a stimulating effect on the patterns. The rise in the SCFAs post sweetener in vitro treatment has been linked to the stimulation of the in vivo transit [20]. Cyclamate and sucralose caused the same metabolomic response, altering the ratio of the butyric and propionic acids when compared to the control sample. A direct relationship was identified between steviol in vitro treatment and the butyrate quantity determined. A significant butyrate value (*p* < 0.001) was recorded after steviol powder was added (Table 3). For this sample, the exception made was the propionic acid synthesis, with about 100 μg/mL less than that of the steviol capsule. These differences can be explained by the presentation of the samples, their dissolution, as well as the presence of other compounds. From the results it is clear that the in vitro treatment of a particular type of sweetener controls the metabolomic response of the microbiota.

**Figure 2:**
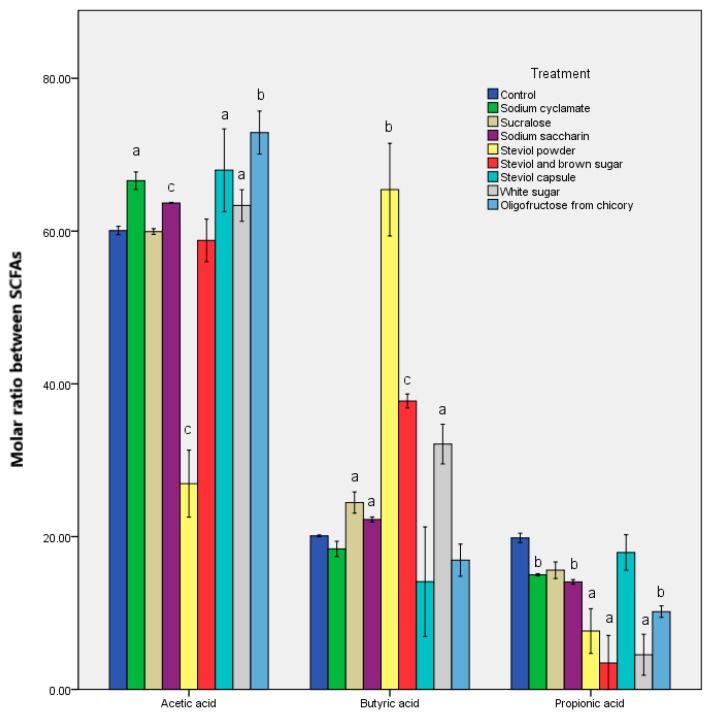
Molar ratio between the acetic, propionic, and butyric acids obtained after the in vitro tests through GIS1 as a measure of the impact of the sweeteners consumed on the metabolic activity of the microbiota. Different letters indicate significant statistical differences (a: *p* ≤ 0.05; b: *p* ≤ 0.01; c: *p* ≤ 0.001), *n* = 3.

The molar ratios of the chief SCFAs are shown in Figure 2 and Figure S1. The microbiological response was the one that generated significant differences in the in vitro treatment of all the sweeteners. The oligofructose from chicory was the exception, where it seemed like a balance was struck compared to the control sample. Steviol led to the most altered values of the molar ratio, while steviol powder led to variations of about three times. These results indicate a change in the metabolomic response even with steviol [21]. There was also an almost 10-fold decrease in the propionate after in vitro steviol treatment. The data revealed a rise in the acetate/butyrate quantities, depending on the type of sample presentation (Table 3).

Another microbiological response to the in vitro treatment of sweeteners was the raised benzoic acid, PLA concentrations, and the HO-PLA in the culture media containing steviol powder, steviol and brown sugar, and white sugar. 

Bifidobacteria and lactobacilli produced significant quantities of PLA and HO-PLA in vitro, using phenylalanine and ά-ketoglutarate [22]. These compounds appear to play a crucial part as antimicrobial and antioxidant compounds [23,24]. Apart from these metabolic compounds, benzoic acid released by the *Serratia marcescens* has been found to play a role in inhibiting the formation of reactive oxygen species in the neutrophils [22]. 

The higher concentrations of these compounds in the samples post sweetener treatment may have been caused by the multiplication of the bacterial populations that synthesize them or by the presence in the environment of certain compounds that stimulate the synthesis of these metabolic compounds. Other organic acids (e.g., oxalic, succinic, malic, tartaric, and citric acids) were not determined after in vitro tests.

### 3.2. Alterations in the Microbiota Pattern Post Sweetener In Vitro Treatment

**Figure 3:**
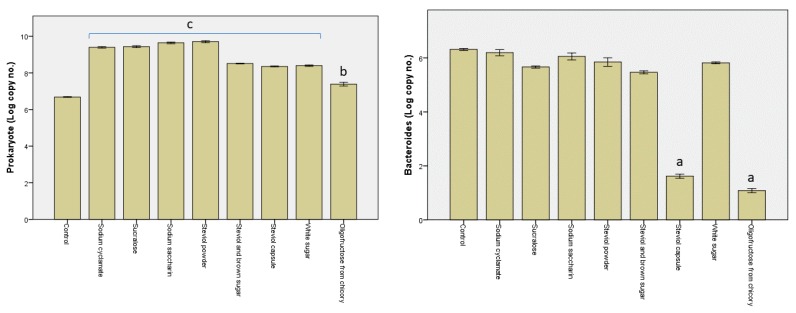

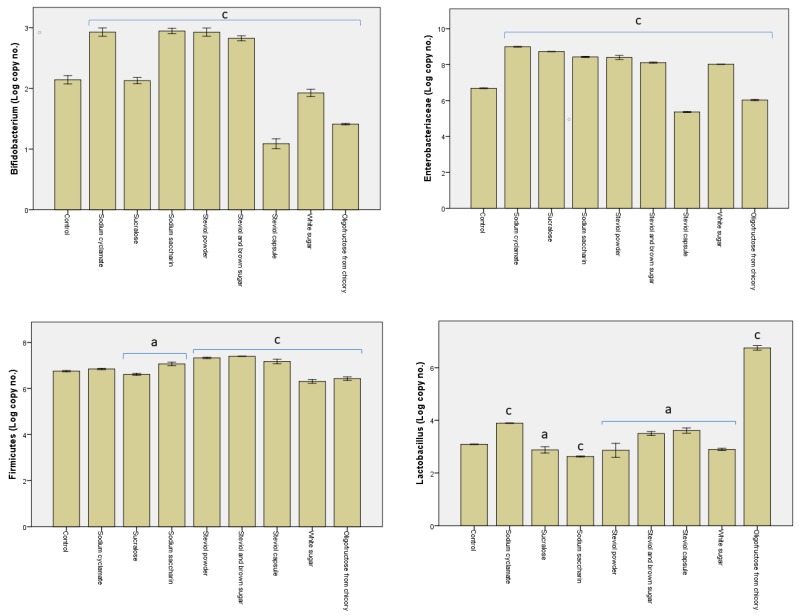
Log of the number of copies obtained post the in vitro tests through the GS1 system as a measure of the impact of the sweeteners on the pattern of the microbiota. Different letters indicate significant statistical differences (a: *p* ≤ 0.05; b: *p* ≤ 0.01; c: *p* ≤ 0.001), *n* = 3.

From the findings shown in Figure 3, significant differences were evident between the samples containing different sweeteners. Considering the general bacterial cells, the highest values were obtained for the sodium cyclamate, sucralose, sodium saccharin, and steviol powder samples (10^9^ genomes/mL), and the lowest value for the oligofructose from chicory sample (10^7^ genomes/mL). For the steviol and brown sugar, steviol capsule, and white sugar samples, however, the number of bacterial cells achieved 10^8^ genomes/mL.

In most samples, the enterobacteria revealed about 10^8^ genomes/mL, barring the steviol capsule and oligofructose from chicory samples, where they decreased, respectively, to 10^5^ genomes/mL and to 10^6^ genomes/mL. In addition, the *Bacteroides–Prevotella–Porphyromonas* groups revealed lower values in these two samples, achieving the minimum detection threshold (10^1^ genomes/mL).

Species included within phylum Firmicutes occurred in large numbers, particularly for sodium saccharin, steviol powder, steviol and brown sugar, and steviol capsule, while their numbers decreased by one unit in samples sodium cyclamate, sucralose, white sugar, and oligofructose from chicory. In the case of the oligofructose from chicory sample, the large number of species within phylum Firmicutes could be linked to the development of the *Lactobacillus–Leuconostoc–Pediococcus* species, where their numbers were roughly equal, achieving 10^7^ genomes/mL. *Bifidobacterium sp.* were found to be low in number in all the samples analyzed, registering below 10^2^ genomes/mL.

## 4. Discussion

The main goal of this study was accomplished by demonstrating the effects of different sweeteners on the human microbiota pattern. One of the most significant findings was the dramatic drop in the number of bifidobacteria after adding the steviol capsule, white sugar, and oligofructose from chicory (Figure 3). The results showed similarity to the data drawn from colorectal cancer patients, and revealed a direct link between the synthesis of SCFAs and modulation of the microbial pattern [25]. When the steviol samples were added in powder form alone or combined with brown sugar (steviol powder and steviol and brown sugar), the number of bifidobacteria was higher than in the control or in the other samples. These results suggest that steviol products could be used as a carbon source by these strains. Thus, when steviol and brown sugar were consumed, the pH of the medium declined (pH < 5). This behavior was characteristic of the descending colon segments, which contained a high number of lactic bacteria. The pH drop was accompanied by the presence of different organic acids (e.g., acetic and lactic acids) in higher amounts (Table 3) upon the administration of steviol powder plus brown sugar (*p* ≤ 0.05) and white sugar. Sweeteners produced by chemical synthesis caused the pH values to increase (>7.5) for saccharin and sucralose (*p* ≤ 0.05; Figure S2). Reports revealed an increase in the number of Gram-negative bacteria—coliforms in particular—which negatively affected the microbiota balance.

Sucralose and sodium saccharin caused a decrease in the number of genomes belonging to Firmicutes, which had a direct correlation with the SCFA level. This behavior was reported in an earlier study, which established the impact of various antibiotics on the SCFAs and precursors of biomarkers [26]. The in vivo effect bears similarity to sucralose in vitro treatment, a compound that can induce the development of inflammatory processes [27]. This finding has been linked to disturbances in the normal microbial pattern, which caused dysbiosis and some alterations in a few physiological functions. This behavior precedes the development of degenerative pathologies [28]. Changes in the microbiota patterns trigger glucose intolerance—one of the stimuli causing type 2 diabetes [29]. Modification of the microbiota pattern corresponds to alterations in the glucose tolerance because the *Bacteroides* species are significant in metabolism regulation [9]. The establishment of dysbiosis was attributed to the rise in the number of the coliform strains that induced the pH to increase and high resistance to long-term modulation of the microbiota [9,29].

By using an improved in vitro static model, the steviol-based sweeteners were shown to have a similar effect to that resulting from prebiotic in vitro treatment. However, no negative physiological changes were observed [30]. From these data (Figure 2 and Figure S1), it is obvious that the in vitro treatment with the steviol-containing products mirrored that of fiber consumption (oligofructose from chicory contains approximately 60% fiber). For the in vitro steviol capsule treatment, the propionic acid/butyric acid ratio [31] was balanced, and similar was seen with oligofructose from chicory. For the other samples, an increase in the butyrate concentration was identified as a biomarker to maintain colon health. The resulting values (Figure S1) offer an explanation for a decline in health status after the sweetener in vitro treatment and the occurrence of dysbiosis.

Modification of the metabolomic pattern after in vitro sweetener treatment was the cause of the microbiota modulation. Sweeteners are generally unaffected by the gastrointestinal environment (neither in low pH nor in bile salts) and do not undergo biotransformation [32]. In addition, some sweeteners (e.g., saccharin) are mostly absorbed in the stomach, and our system does not allow us to highlight this process [32]. A reduced metabolic activity of the microbiota was noted. The results of this study did not confirm an earlier in vivo study, which stated that in vitro sucralose treatment induced an alteration in the number of *Enterobacteriaceae* [33]. The differences were understood to be a result of the types of experiments conducted, as well as of the specificity of the tested microbiota. 

The study showed that the microbial load was reduced, even with in vitro steviol treatment (e.g., steviol capsule). This limits the plasticity of the microbial pattern to exogenous factors. In our case, the behavior was due to the selective consumption (use as carbon source) of the sweetener by the lactic bacteria strains [6]. Also, it was supported by acetic acid (Table 3) and lactic acid synthesis, for steviol and brown sugar and white sugar [34]. 

Microbiota control demonstrated that in vitro sweetener treatment caused the fermentation processes to escalate, due to the selective use of these compounds. This behavior was evident for oligofructose from chicory, and the steviol action on the metabolomic profile was confirmed by an earlier study on the effect of fiber on healthy donors [35]. According to earlier studies [36], steviol was shown to be able to enhance the glucose uptake, revealing an effect similar to human insulin. The partially contradictory in vitro data can also be explained by the limitations of using a static simulator. Further, the final pattern revealed a specific signature, affected directly by the carbon source and selective modulation of the microbiota. Steviol significantly raised the quantity of the SCFAs (Table 1), and the phenomenon mirrored the in vivo behavior of obese individuals, where the assimilation of the SCFAs increases the daily calorie intake [37]. The increased quantity of the SCFAs was also related to supporting the physiological function of the colon by promoting an anti-inflammatory response. In vitro steviol treatment data lend support, via the SCFAs content, to the fact that it does not exert any negative effect on the body [38].

This study is relevant when considering large-scale sweetener consumption, by demonstrating their impact on the colon microbiota. The metabolomic modulation by the steviol was demonstrated by the complete metabolism compared to the rest of the samples [6,39]. Some differences were noted between the samples containing steviol, which can be explained as one of the effects of product presentation (powder, tablet, or combination with other compounds). Steviol capsule along with oligofructose from chicory determined a significant decrease in the Gram-negative strains, and also in bifidobacteria. The sodium cyclamate, sodium saccharin, steviol powder, and steviol with brown sugar induced an increase in bifidobacteria. The possible presence of other compounds (e.g., carrier ingredients; Table 1) may represent one of the limitations of this study. In our study, one example is sodium bicarbonate, which is present in small quantities in steviol capsules. Though the presence of this ingredient does not have a negative effect, and it is considered to prevent type 2 diabetes incidence, in our research the quantities were too small to express an effect and to have an influence on microbiota activities [40]. The results of the study refer to the effects of these sweeteners on the microbiota starting from the action of the major active compound in the sweetener composition. Other minor compounds (e.g., excipients, carrier ingredients, or the presence of other minor sweeteners) may have a synergistic role, amplifying the effect of the principal active compound [41].

## 5. Conclusions

In conclusion, the study has proved that both the fermentative response and microbial diversity were altered after in vitro sweetener treatment. Non-nutritional sweeteners were found to induce toxicity [42], expressed by the instauration of dysbiosis. Any alteration in the microbial and metabolomic patterns causes physiological dysfunctions which can trigger the incidence of chronic diseases [43]. On the other hand, understanding the effect of sweeteners on some groups of microorganisms from colon microbiota gives us the possibility of modulating the pattern, by supplementing the diet with certain sweeteners. The in vitro steviol treatment induced a rise in the SCFA synthesis, not related to the variations in the genome counts, which limited the physiological response. In the future, determining the antioxidant response to steviol will have to be considered for its use as a nutraceutical and for modulating the metabolomic pattern.

## Figures and Tables

**Table 1:** Sample presentation and composition of the sweeteners.

Samples Composition	Producer
Sodium cyclamate	KClassic Sweetener
Sucralose	Carrefour Quality BE
Sodium saccharin	Biscol LTD, Israel
Steviol powder	Kruger & Co., Germany
Steviol and brown sugar (1:1)	Tate & Lyle Sugars, Tesco, UK
Steviol capsule	Sly Nutricia SRL, Romania
White sugar	Sugar Factory Diamant, Romania
Oligofructose from chicory	Mărgăritar Sweet & Fit, AGRANA Zucker A.G. Vaslui, Romania

**Table 2:** Primer sequences and conditions used in the qPCR reaction.

Groups	Sequence: 5′-3′		Primer Conc.	Slope	Efficiency (%)	Reference
Prokaryote	F	CGG YCC AGA CTC CTA CGG G	0.2 µM	−3.22	104.16	[15]
	R	TTA CCG CGG CTG CTG GCA C				
*Lactobacillus–Leuconostoc–Pediococcus* Group	F	AGC AGT AGG GAA TCT TCC A	0.5 µM	−3.51	92.70	[16]
	R	CAC CGC TAC ACA TGG AG				
*Bifidobacterium sp.*	F	TCG CGT CYG GTG TGA AAG	0.3 µM	−3.49	93.38	[16]
	R	CCA CAT CCA GCR TCC AC				
*Enterobacteriaceae* Family	F	CAT TGA CGT TAC CCG CAG AAG AAG C	0.3 µM	−3.39	97.17	[15]
	R	CTC TAC GAG ACT CAA GCT TGC				
*Bacteroides–Prevotella–Porphyromonas* Group	F	GGTGTCGGCTTAAGTGCCAT	0.3 µM	−3.32	99.68	[16]
	R	CGGAYGTAAGGGCCGTGC				
Firmicutes Phylum	F	GGAGYATGTGGTTTAATTCGAAGCA	0.5 µM	**−**3.28	101.52	[17]

**Table 3:** Organic acid levels (µg/mL) obtained after the in vitro tests through GIS1 as a measure of the impact of the sweeteners consumed on the metabolic activity of the microbiota.

Samples	Formic Acid	Lactic Acid	Benzoic Acid	Phenyllactic Acid	HO-Phenyllactic Acid
Control	34.21 ± 5.35	nd	1.71 ± 0.10	17.60 ± 0.36	44.58 ± 0.76
Sodium cyclamate	49.63 ± 3.70 ^c^	nd	1.71 ± 0.05 ^a^	24.19 ± 0.18 ^b^	48.81 ± 0.89 ^a^
Sucralose	41.29 ± 3.40 ^b^	nd	1.60 ± 0.07 ^a^	38.50 ± 1.99 ^c^	56.48 ± 1.57 ^b^
Sodium saccharin	32.13 ± 1.18 ^a^	nd	1.34 ± 0.1 ^c^	28.86 ± 1.02 ^a^	53.49 ± 1.36 ^b^
Steviol powder	12.68 ± 0.98 ^a^	nd	7.87 ± 0.15 ^b^	179.22 ± 4.46 ^c^	72.96 ± 1.36 ^b^
Steviol and brown sugar	46.85 ± 1.96 ^c^	314.43 ± 5.89 ^b^	12.06 ± 0.12^c^	120.35 ± 4.15 ^a^	41.83 ± 1.07 ^c^
Steviol capsule	nd	nd	2.74 ± 0.09 ^a^	42.52 ± 0.52 ^b^	118.15 ± 1.40 ^a^
White sugar	313.51 ± 8.56 ^b^	291.52 ± 5.60 ^a^	23.66 ± 0.23 ^a^	8.68 ± 1.29 ^a^	141.00 ± 0.24 ^c^
Oligofructose from chicory	435.74 ± 3.93 ^c^	nd	3.90 ± 0.13 ^c^	55.96 ± 0.75 ^c^	42.38 ± 0.17 ^b^

Different letters mean significant statistical differences (a: *p* ≤ 0.05; b: *p* ≤ 0.01; c: *p* ≤ 0.001), *n* = 3; nd: not determined.

## Supplementary Materials

The following are available online at https://www.mdpi.com/2073-4425/10/7/535/s1. Figure S1: SCFAs levels (μg/mL) obtained after the in vitro tests through GIS1, Figure S2: pH values obtained during the in vitro tests (descending segment) through GIS1.
